# Construction of a Prognostic Model based on CSC-related Genes in Patients with Colorectal Cancer

**DOI:** 10.7150/jca.108188

**Published:** 2025-04-13

**Authors:** Zi-Yue Li, Ming-Feng Li, Ying-Ying He, Guan-Sheng Zheng, Jie-Rong Chen, Yun-Miao Guo, Qizhou Lian, Cai-Feng Yue

**Affiliations:** 1 Zhanjiang Institute of Clinical Medicine, Central People's Hospital of Zhanjiang, Guangdong Medical University Zhanjiang Central Hospital, Zhanjiang 524045, China.; 2 Cord Blood Bank, Guangzhou Institute of Eugenics and Perinatology, Guangzhou Women and Children's Medical Center, Guangzhou Medical University, Guangzhou 510623, China.; 3 Department of Anesthesiology, Central People's Hospital of Zhanjiang, Guangdong Medical University Zhanjiang Central Hospital, Zhanjiang 524045, P. R. China.; 4 Department of Laboratory Medicine, Guangdong Provincial People's Hospital (Guangdong Academy of Medical Sciences), Southern Medical University, Guangzhou 510080, China.; 5 Department of Laboratory Medicine, Central People's Hospital of Zhanjiang, Guangdong Medical University Zhanjiang Central Hospital, Zhanjiang 524045, China.

**Keywords:** Colorectal cancer, Cancer stem cells, Prognosis, Tumor microenvironment, Immune checkpoint inhibitors

## Abstract

Colorectal cancer (CRC) is one of the most common and deadly malignancies. Lack of efficient biomarkers for prognosis has limited the improvement of survival outcome in patients with CRC. Numerous studies have demonstrated the important roles of cancer stem cells (CSCs) in both treatment resistance and disease recurrence of CRC. Thus, the current study aims to construct a prognostic model based on expression level of CSC-related genes for precise molecular subtyping of CRC patients with different prognoses, TME infiltration patterns and therapeutic responses. The RNA sequencing data and clinical information were obtained from UCSC Xena database, followed by identification of differential expressed genes, univariate Cox regression, and LASSO regression to identify prognostic CSC-related genes and construct a novel prognostic risk scoring model consisting of 21 CSC-related genes. The patients in high-risk group suffered poor survival outcome (*P*<0.0001). Moreover, the performance of CSC-related prognostic model was validated in individual GEO datasets including GSE41258 and GSE39582 (*P*<0.05). Furthermore, patients with high-risk score exhibited lower response rate to immune checkpoint inhibitors as compared to those in low-risk group (17.4% vs. 28.2%), indicating the potential of CSC-related prognostic model to predict the immunotherapy response. Collectively, our findings provide an effective model to predict the immunotherapy response and survival outcome in patients with CRC.

## Introduction

Colorectal cancer (CRC) is currently the third most common malignancy and the second leading cause of cancer-related mortality worldwide, with 1,931,590 newly diagnosed cases and 935,173 deaths from cancer in 2020^1^. The long-term outcomes of patients with CRC have substantially improved due to considerable evolvement of surgical treatment, chemotherapy, and immunotherapy^2^. However, approximately one-fourth of patients present with distant metastases at the time of diagnosis and additional 25-50% of patients diagnosed at early stages subsequently develop metastatic diseases, which are major causes of treatment failure and thus poor prognosis^3^. Therefore, there is an urgent need to identify effective biomarkers or indicators for treatment guidance and prognosis prediction in patients with CRC.

Cancer stem cells (CSCs) are a small subset of cancer cells with the ability to self-renew and dedifferentiate, which are critical for initiating and sustaining the growth of tumor^4^. The aberrant expression of CSC-related genes is supposed to play important roles in regulating the proliferation, metastasis, and therapeutic resistance of tumor cells, and thus shed a new light on the CSC-targeted therapies of tumor^5^. Recently, the gene expression-based stemness index (mRNAsi) was utilized for identification of therapeutic targets and precise prognosis in multiple cancers including gastric cancer^6^,^7^, head and neck squamous cell carcinomas^8^,^9^, prostate adenocarcinoma^10^, esophageal cancer^11^, bladder cancer^12^, lung cancer^13^, and glioma^14^. Collectively, the findings have revealed the potential of CSC-related genes as biomarkers to satisfy the unmet need for risk stratification and treatment optimization in patients with cancer. However, the relationship between the CSC-related genes and outcome in patients with CRC has been rarely explored.

In the current study, a prognostic model based on expression level of CSC-related genes was established for precise treatment planning and accurate prognosis of patients with CRC.

## Materials and Methods

### Data acquisition

The gene-level copy number data (SNP6.0 array), DNA methylation (Methylation 450K array), mRNA and miRNA expression data (z-score normalized), list of somatic mutations (including SNPs and INDELs) and copy number variations (CNV, including AMP and DEL), reverse phase protein array (RPPA) data, stemness scores (DNA methylation based and RNA expression-based), immune signature scores, and corresponding phenotype data of TCGA Pan-Cancer (PANCAN) cohort were collected by using UCSC Xena^15^. The different sets of transcript expression data were re-calculated and normalized by using UCSC TOIL recompute pipeline. After exclusion of subjects diagnosed under age 18, a total of 12,591 subjects and 33 cancer types were enrolled for further analyses. In the TCGA-COAD cohort, patients with age under 18, relapsed/secondary tumors, ambiguous and/or missing clinical and follow-up data were excluded. Eventually, a total of 450 patients were included for subsequent analyses. Moreover, eight expression profile datasets including GSE13507, GSE4412, GSE21653, GSE41258, GSE84437, GSE42127, GSE23554, GSE57495, and GSE39582 were downloaded from Gene Expression Omnibus (GEO) database^16^ as validation sets. Furthermore, the clinical characteristics and RNA expression data of patients with urothelium carcinoma in IMvigor 210 cohort were downloaded by using R package IMvigor210CoreBiologies to assess the response to immunotherapy^17^. Responders are referred to as patients with complete remission (CR), or partial remission (PR), whereas non-responders are defined as those with stable disease (SD) or progressive disease (PD). The infiltration of immune cells and response to therapies were evaluated by using CIBERSORT^18^ algorithm, ESTIMATE^19^ algorithm, TIDE^20^ algorithm, and GDSC^21^ database, respectively. The list of CSC-related genes was obtained by searching in the molecular signatures database (MSigDB)^22^, cancer stem cells database (CSCdb)^23^, and published literatures.

### Classification of molecular subtypes

CSC-related genes associated with survival of cancer patients were identified by univariate Cox regression analysis, followed by consensus clustering using R package ConsensusClusterPlus^24^. The relative change in area under the CDF curve was evaluated to determine the optimal k value and thus the number of clusters. The difference in length of survival time between distinct molecular subtypes was assessed by weighted log-rank test, and Kaplan-Meier (K-M) curves were plotted by using R package survival. The hazard ratio (HR) and ***P*** were calculated by using Cox regression analyses between molecular subtypes with the most favorable or poorest prognosis in multiple cancers, followed by validation in additional GEO datasets.

### Identification of Differentially Expressed Genes (DEGs) among different subtypes

DEGs between molecular subtypes with either best or poorest prognosis were identified by using R package limma^25^ according to the threshold of |log_2_ fold change (FC)| ≥ 1 and false discovery rate (FDR) < 0.05. Subsequently, R packages EnhancedVolcano and pheatmap were employed to visualize the results of differential expression analyses.

### Construction and validation of prognostic model based on CSC-related genes

Candidate genes represented in both lists of DEGs among different subtypes and CSC-related genes were further analyzed by using univariate Cox regression, and genes with ***P*** ≤ 0.01 were identified as prognosis-associated genes. Subsequently, Lasso regression was applied to perform dimensionality reduction and establish the prognostic model. The risk score for each patient with CRC was calculated according to the following formula, in which *C_j_* represents the regression coefficient for gene j and *exp_ij_* represents the expression of gene j in sample i.



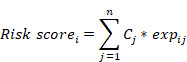



The same formula was used in both the training set and external validation cohorts. Patients were assigned to low-risk or high-risk subset using the median of risk scores as threshold. Kaplan-Meier (K-M) curves and log-rank tests were applied to assess the difference in outcome of patients. R package timeROC was employed to generate ROC curve and calculate area under time dependent ROC curve (AUC). The risk scores of 298 patients from IMvigor210CoreBiologies dataset were calculated to evaluate their predictive ability of immunotherapeutic responsiveness by using Kruskal-Wallis test.

### Statistical analysis

Statistical analyses were carried out by using R (version 4.1.2). Statistical significance between two groups was tested using Student's t-test. For variables more than three groups, a one-way analysis of variance or the Kruskal-Wallis test was used, depending on the type of data. Correlation coefficients were calculated using Spearman's correlation analysis. ***P*** < 0.05 was considered to indicate a significant difference, unless otherwise stated.

## Results

### Identification of Molecular Subtypes based on CSC-related Genes

**Figure 1:**
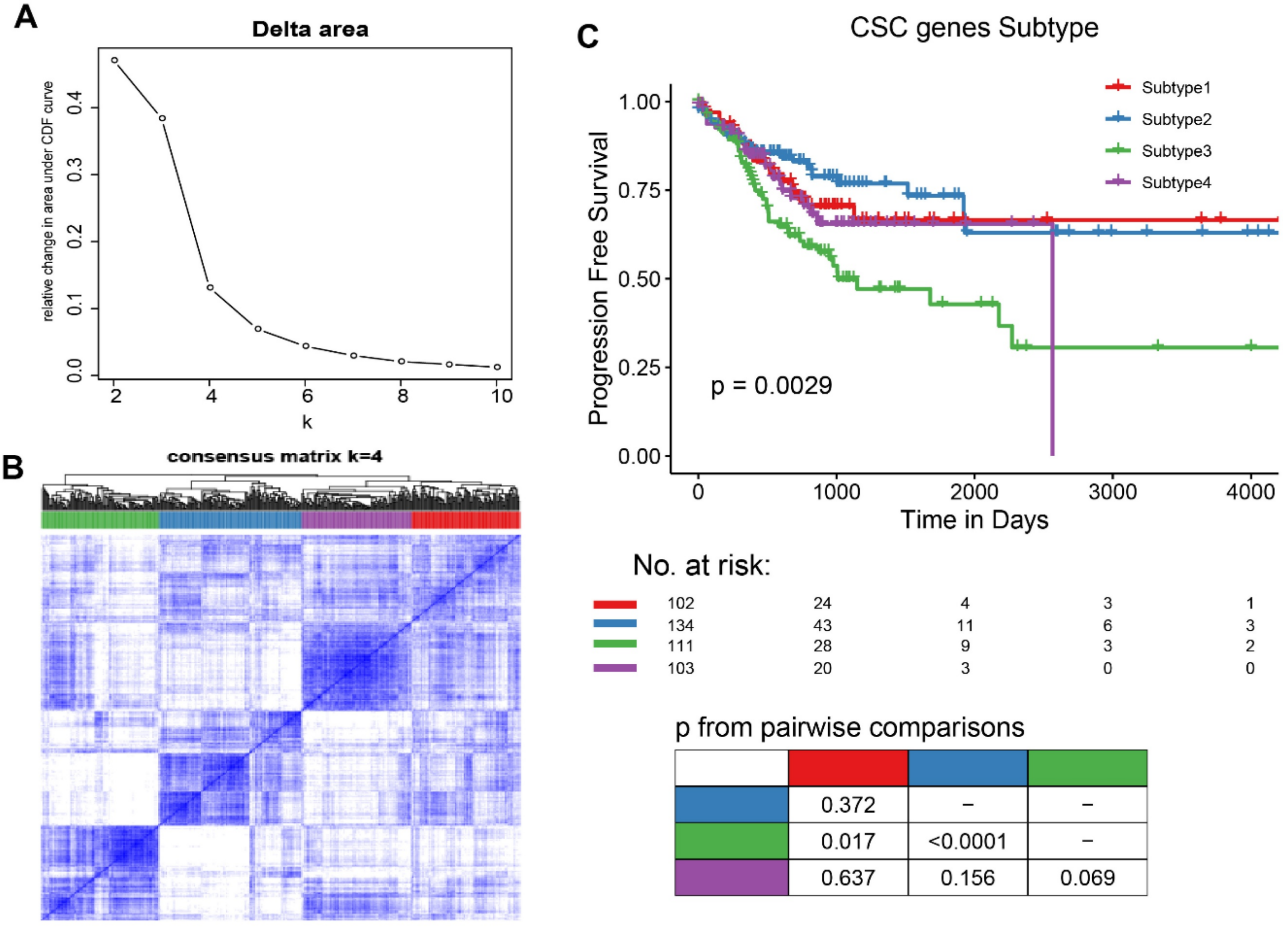
Identification of CSC-related subtypes by K-means analysis. (A-B). K = 4 was identified as the optimal value for consensus clustering, the patients were divided into 4 distinct gene clusters. C. Kaplan-Meier survival curve showing survival probability for the 4 subtypes.

Thirty-four out of 206 selected CSC-related genes were differentially expressed in CRC tissues as compared with normal tissues, as well as in other cancers cataloged in TCGA database (Figure S1, Supporting Information). On the basic of the expression level of 34 differentially expressed genes (DEGs), patients with CRC were divided into four molecular subtypes with different lengths of progression free survival time (Figure 1A-B), patients in subtype 2 had significantly better clinical outcome than those in subtype 3(Figure 1C). Furthermore, the differences in survival outcome were observed between subtypes classified based on the expression of DEGs in patients with diverse types of cancer in TCGA database (Figure S2A, Supporting Information) and GEO datasets (Figure S2B, Supporting Information).

### Characteristics of CSC-related clusters for COAD

**Figure 2:**
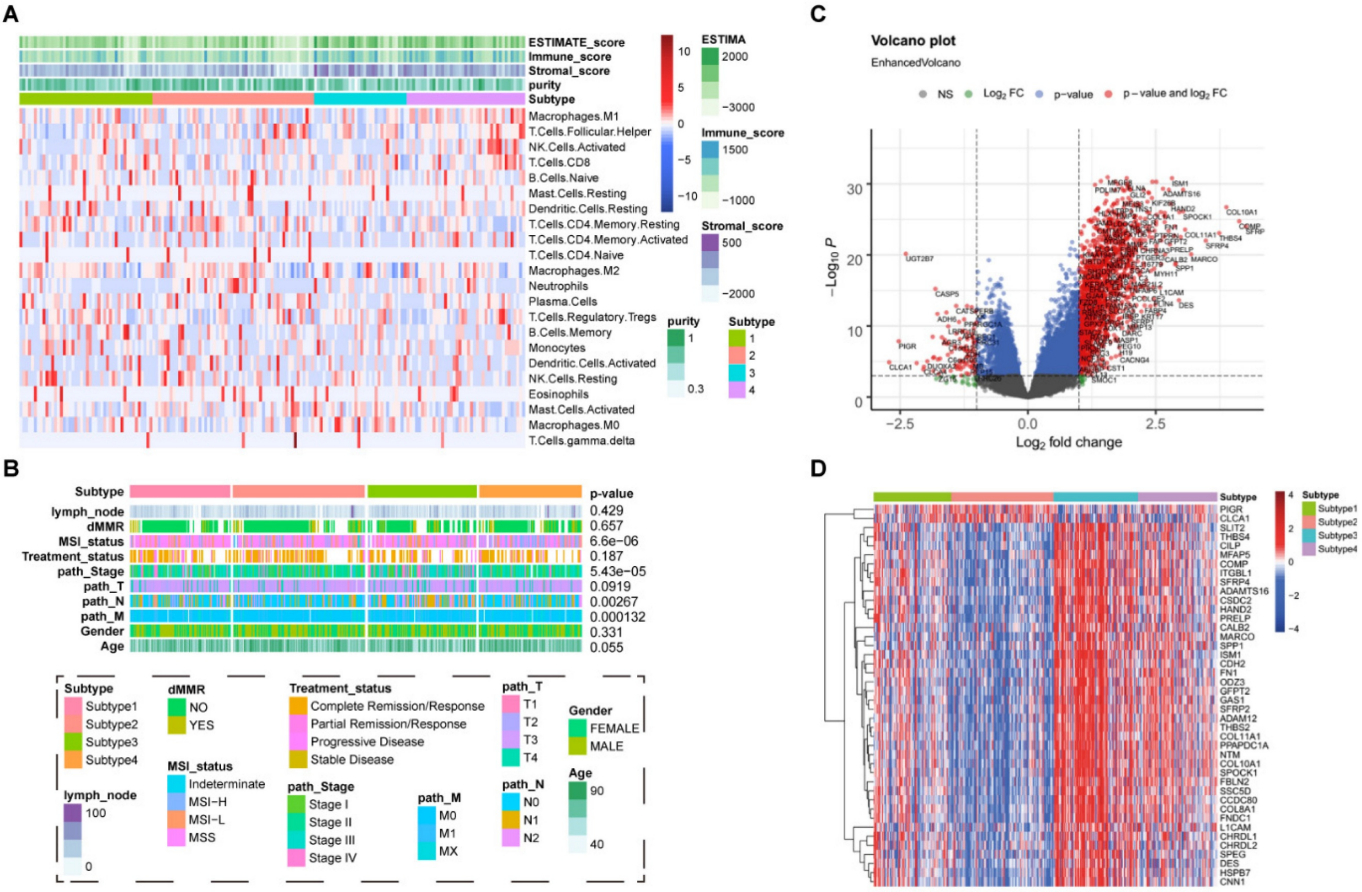
Characteristics of CSC-related clusters for COAD. (A). The immune infiltration levels of 22 immune cells among the 4 subtypes in COAD. (B). Heatmap showing the 4 subtypes in different clinical characteristics and clusters. (C). The volcano map reflects the differential expressed genes identified (|Log_2_FC| >1 and ***P*** <0.05). (D). Heatmap showing the top 50 differential expressed genes among the 4 subtypes.

To explore the characteristics of CSC-related clusters, immune infiltration levels of 22 immune cells among the 4 subtypes in COAD were obtained from known studies and shown in Figure 2A. Moreover, clinical characteristics including the number of lymph nodes, whether MMR is deficient (dMMR), MSI statues treatment statues, pathological stage, TNM stage, age, and gender were interrogated among different subtypes (Figure 2B). A total of 1065 differentially expressed genes (DEGs) were identified (|log_2_FC| ≥ 1, ***P***< 0.05), and the volcano map accurately reflected the gene expression differences between subtype 2 and subtype 3 (Figure 2C). The top 50 differentially expressed genes among different subtypes was shown in heatmap (Figure 2D).

### Construction of CSC-related genes signature for COAD

**Figure 3:**
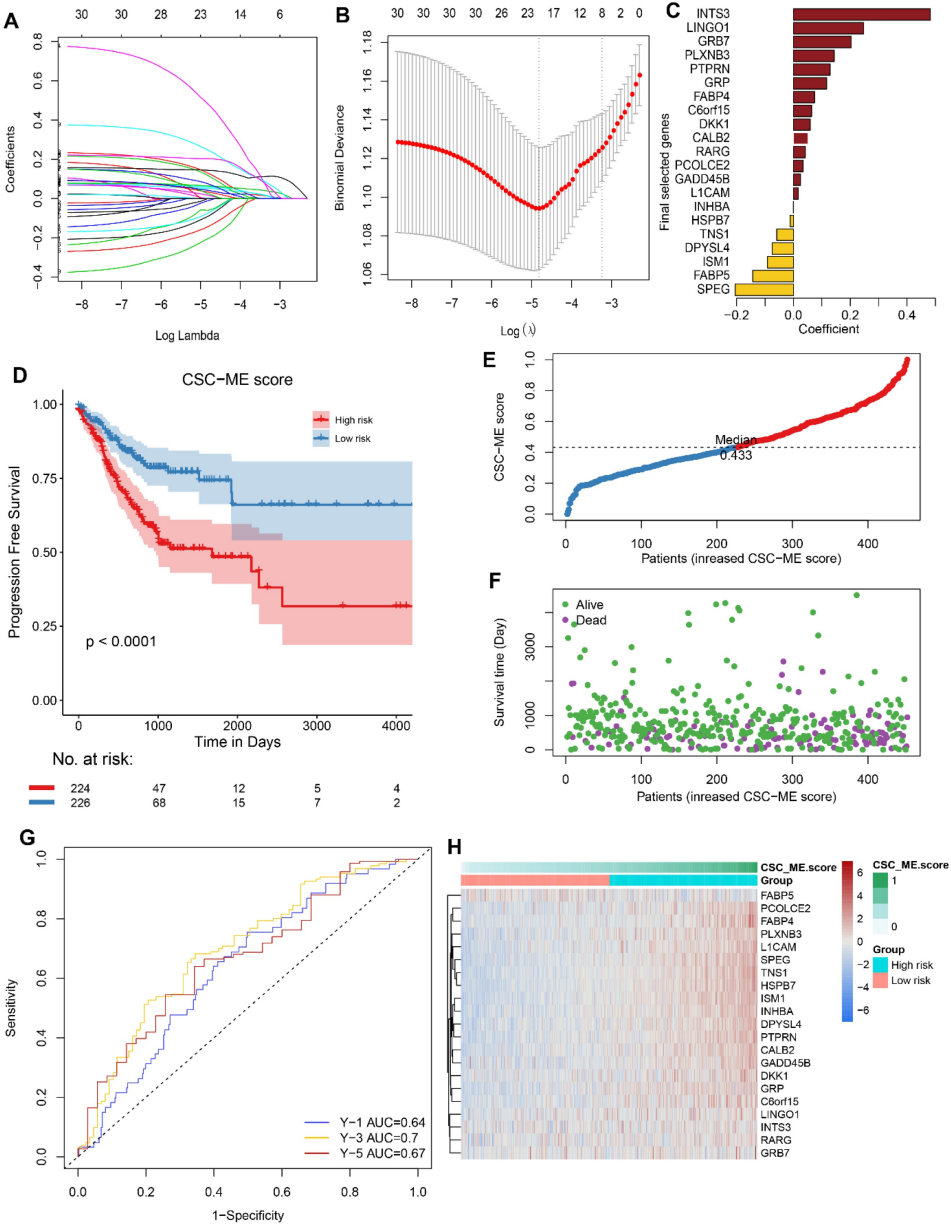
Construction of CSC-related genes signature for COAD. (A, B) The LASSO regression analysis of CSC-related genes associated with prognosis. (C). The coefficient score of the final selected genes. (D). Kaplan-Meier survival curve showing survival probability of high-risk or low-risk subgroups. (E). Risk score curve plot. The dotted line indicates the individual distribution of risk score, and the patients are categorized into low-risk (blue) and high-risk (red) groups. (F). Risk score scatter plot. Purple dots indicate the dead patients, and green dots indicate the alive. With the increase in risk score, more patients died. (G). The 1-year, 3-year, and 5-year survival ROC curves are predicted by the signature. (H). Heatmap showing the distribution of 21 genes in the prognostic signature between the two groups.

The differential expressed genes were combined with CSC-related genes. After Univariate Cox regression analysis and least absolute shrinkage and selection operator (LASSO) regression analysis (Figure 3A-B), 15 potential pro-oncogenes (HR > 1, *INTS3, LINGO1, GRB7, PLXNB3, PTPRN, GRP, FABP4, C6orf15, DKK1, CALB2, RARG, PCOLCE2, GADD458, L1CAM, INHBA*) and 6 potential suppressor genes (HR < 1, *HSPB7, TNS1, DPYSL4, ISM1, FABP5, SPEG*) were identified (Figure 3C). The above CSC-related genes were used to develop the risk score prognostic signature, and the risk score for each COAD sample was calculated according to the following formula: coefficient × Expr (*INTS3, LINGO1, GRB7, PLXNB3, PTPRN, GRP, FABP4, C6orf15, DKK1, CALB2, RARG, PCOLCE2, GADD458, L1CAM, INHBA, HSPB7, TNS1, DPYSL4, ISM1, FABP5, SPEG*). Patients were divided into high-risk and low-risk groups according to the median risk score. The high-risk group had significant worse clinical outcomes (PFS: *P*<0.0001, Figure 3D; OS: *P*=0.0038, Figure S3, Supporting Information). Risk score curve plot and curve plot were shown in Figure 3E-F. The survival ROC curves predicted by the signature showed that the AUCs were all greater than 0.8, indicating the effectiveness of the CSC-related signature in predicting prognosis for COAD at the 1-year (AUC=0.64), 3-year (AUC=0.7), and 5-year (AUC=0.67) time points (Figure 3G). Heatmap displayed the distribution of 21 genes in the prognostic signature between the two groups (Figure 3H).

### Validation of the prognostic signature in GEO Cohort

**Figure 4:**
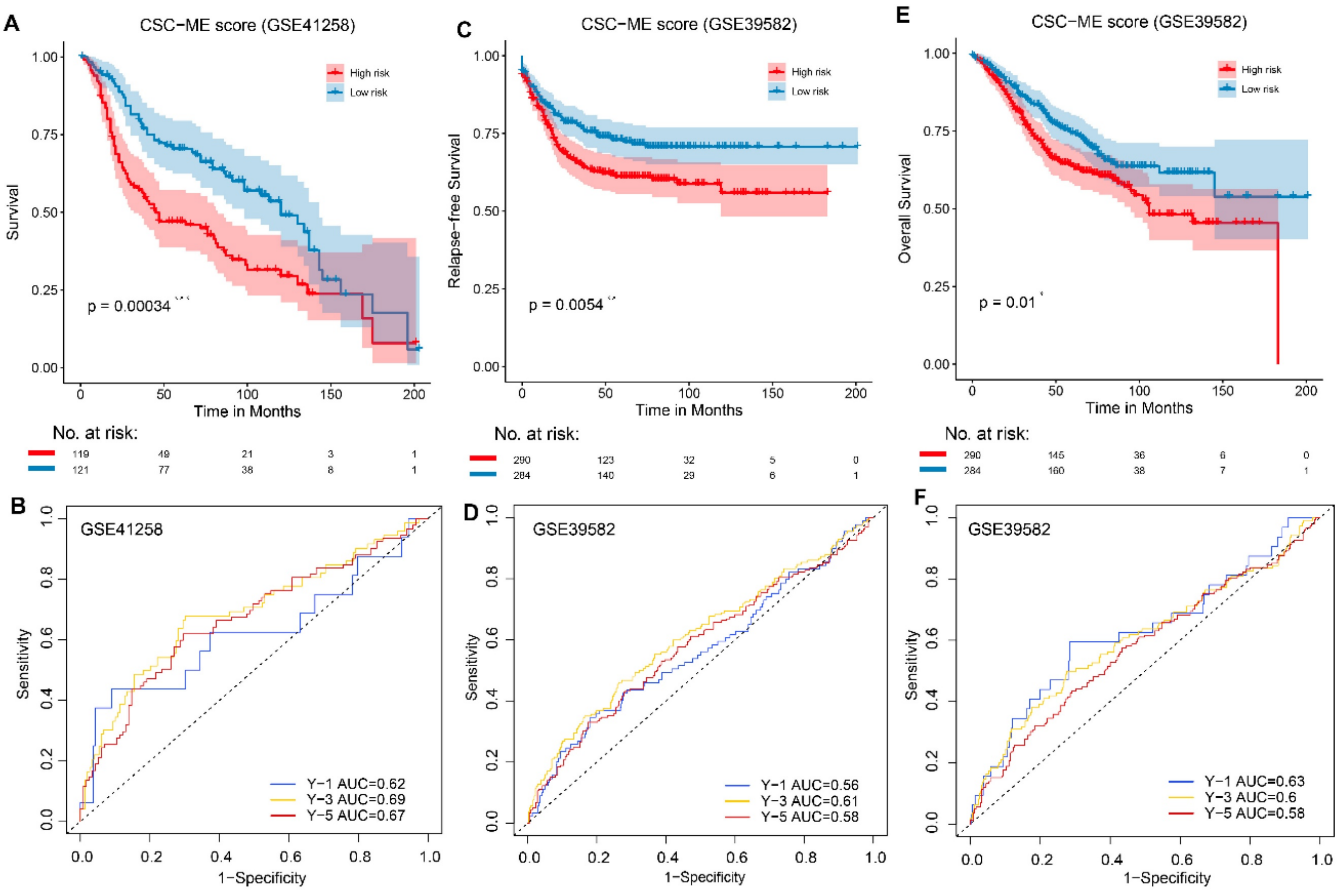
Validation of the constructed prognostic signature in GEO cohorts. (ACE, *, ** and *** stands for ***P*** < 0.05, 0.01 and 0.001 respectively). Kaplan-Meier survival curve showing survival probability of high-risk or low-risk subgroups. (BDF). The 1-year, 3-year, and 5-year survival ROC curves are predicted by the signature.

To validate the performance of the CSC-related signature in predicting OS, risk scores were calculated with the same formula for patients in GSE41258 and GSE39582. Similarly, the survival curve in GEO cohort also demonstrated that the high-risk group showed a poor overall survival compared to the low-risk group (Figure 4). Moreover, the survival ROC curves showed good effectiveness in predicting prognosis (Figure 4).

### Immunotherapy response prediction

**Figure 5:**
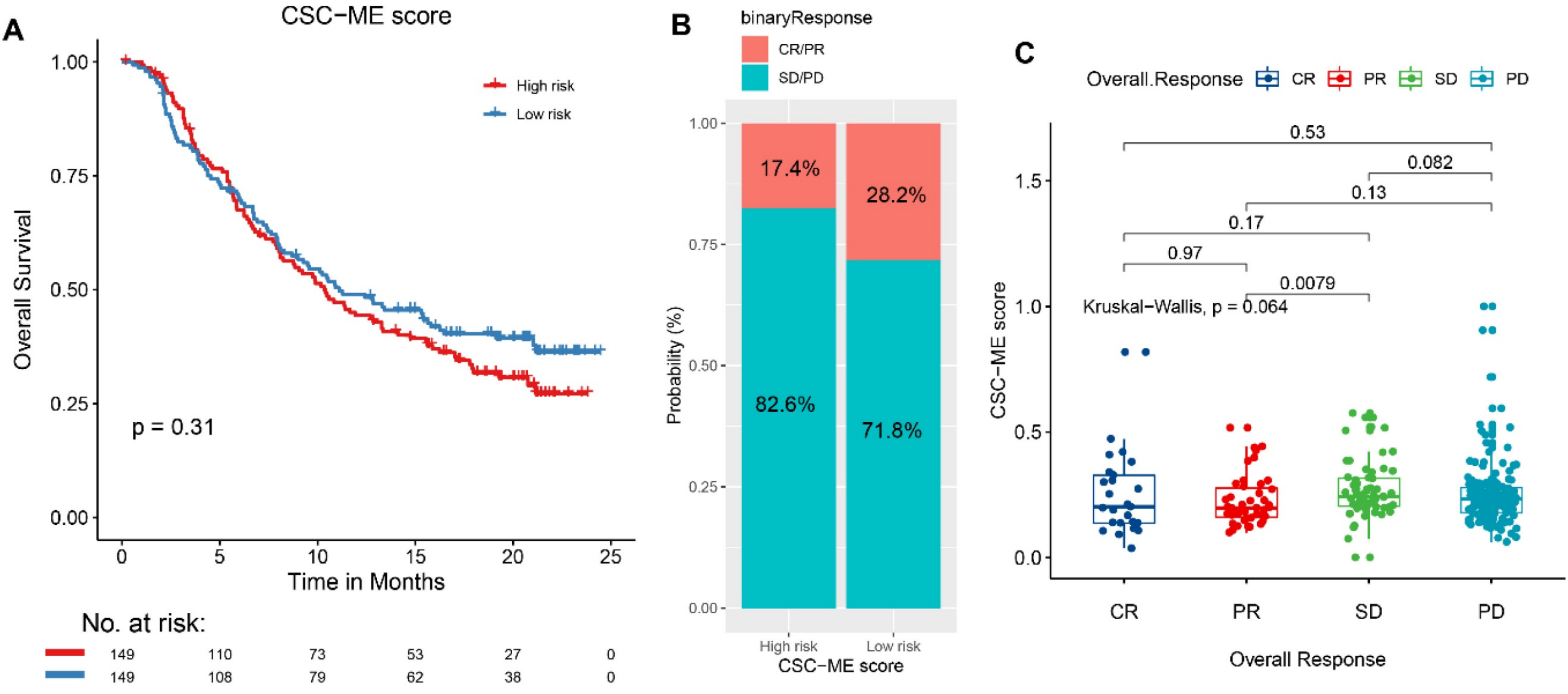
A high-risk score predicts poor response to immune checkpoint inhibitors (ICIs). (A). Overall survival (OS) analysis of high-risk and low-risk groups. (B). Comparison of Immunotherapy response ratio between high-risk and low-risk groups. (C). Comparison of risk scores between different immune response states.

The results based on the use of the Imvigor210CoreBiologies dataset showed that patients in the high-risk group exhibited no adverse OS compared to those in the low-risk group (***P*** = 0.31, log rank test; Figure 5A). However, the response rate to ICIs was significantly higher in the low-risk group than that in the high-risk group (28.2% vs. 17.4%, respectively; Figure 5B). Concurrently, non-responders to ICIs (SD + PD) presented with higher risk scores than responders (CR + PR, Figure 5C). This finding indicates that the risk score can be used as a prognostic marker of the immune response.

### The landscape of CSC-related score in pan-cancers

**Figure 6:**
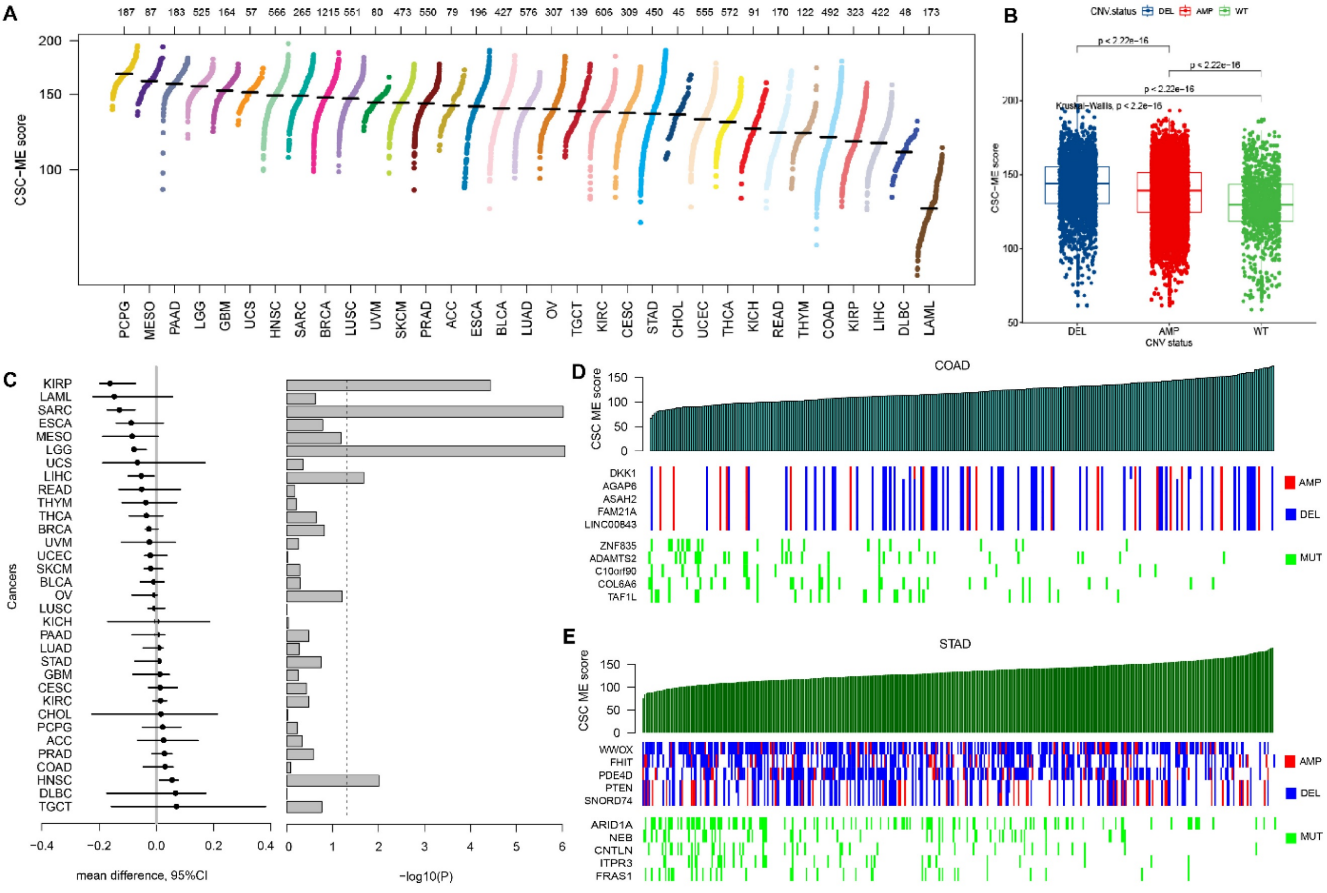
CSC-related score in pan-cancer and the relation with CNV. (A). Scatter plot of CSC-related score in pan-cancer. (B). Boxplot of samples with CNV (including AMP and DEL) versus wild type (WT). (C). Relative difference value and significance distribution of samples with CNV in pan-cancer. Genes with significant CNV and genes with somatic mutations in COAD (D) and STAD (E) as CSC-related score increased.

The CSC-related score was calculated among all types of cancers and shown in Figure 6A. Samples with CNV had significantly higher CSC-related score than those without (Figure 6B). The CSC-related score showed a correlation with CNV in pan-cancers (Figure 6C). For example, the KIRP patients with AMP had a significant higher CSC-related score. Meanwhile, the HNSC patients with DEL had a significant higher CSC-related score. Genome-wide variation with CNV and somatic mutation was shown as the CSC-related score increased in GI cancers, including COAD (Figure 6D) and STAD (Figure 6E).

### Survival analyses of CSC-related signature in pan-cancers

**Figure 7:**
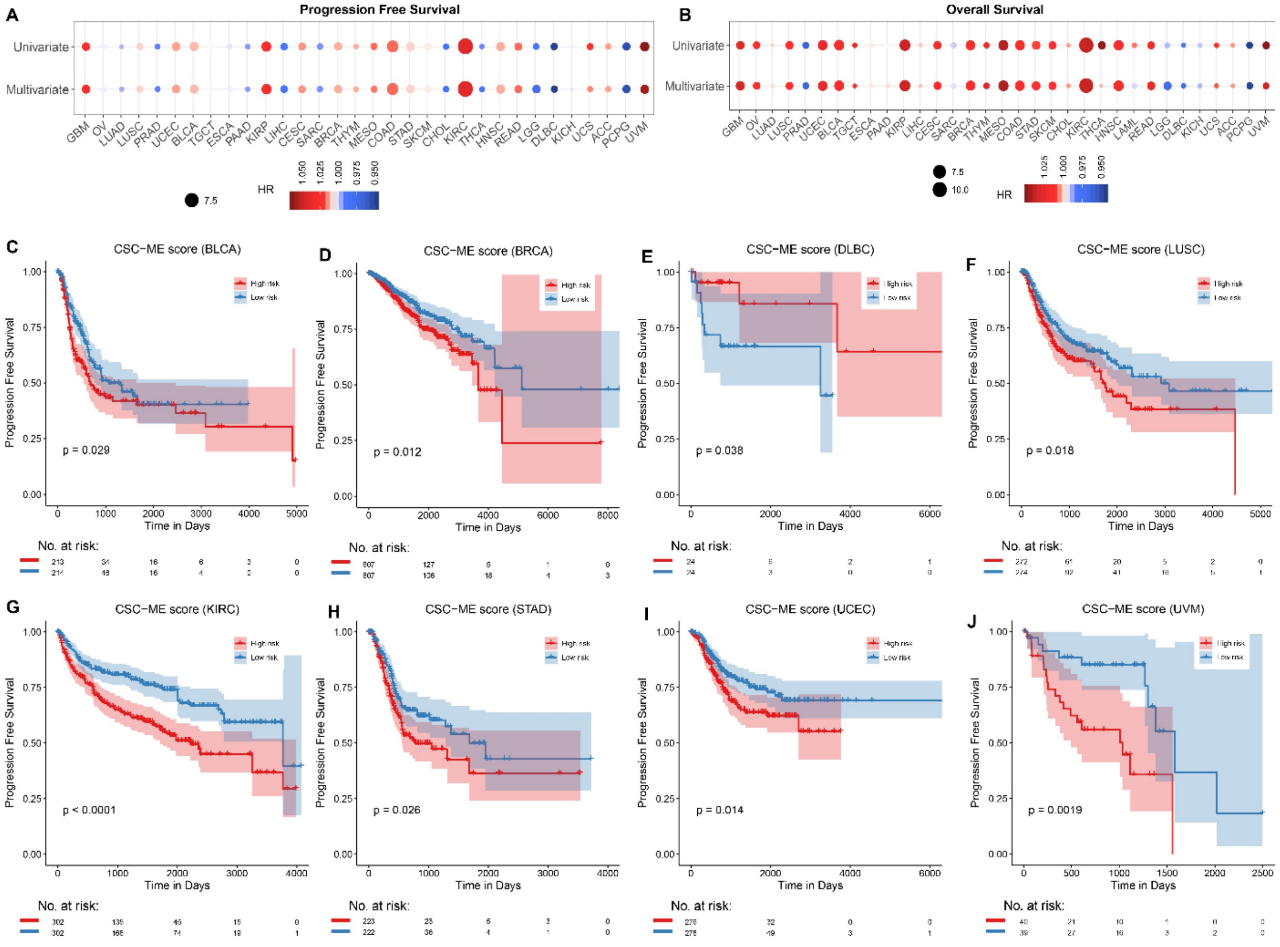
Survival analyses of CSC-related signature in pan-cancers. Univariate and multivariate Cox regression analysis of CSC-related score on the risk of PFS (A) and OS (B) in pan-cancer. (C-J). Survival curves (PFS) of high-risk and low-risk groups in pan-cancer.

Univariate Cox regression analysis and multivariate Cox regression analysis (adjusted for age, gender, and tumor grade) were applied to calculate the risk of CSC-related score on patient survival time (including PFS and OS, Figure 7A-B). The samples were divided into high-risk and low-risk groups according to the median CSC-related score. Kaplan-Meier curves for progression-free survival (PFS, Figure 7C-J) and overall survival (OS, Figure S4, Supporting Information) in pan-cancers were significant, the low-risk group had a higher survival rate.

### Correlation of CSC-related score with immune characteristics and stemness score in pan-cancer

**Figure 8:**
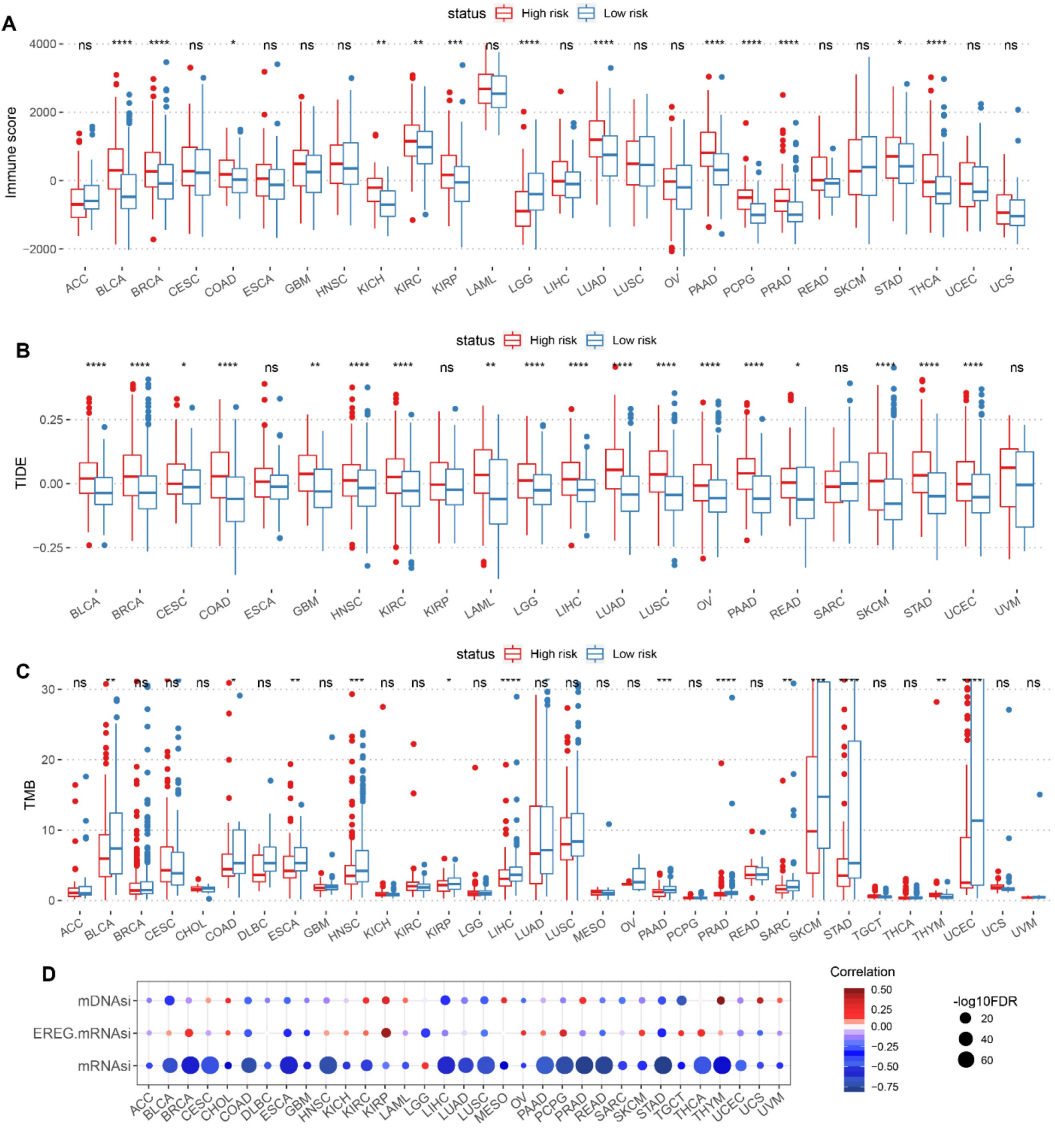
Correlation of CSC-related score with immune characteristics and stemness score in pan-cancer. Boxplot displaying immune characteristics including immune score (A), TIDE score (B), and TMB (C) between high and low CSC-related score groups. (D) Correlation of CSC-related score with mDNAsi, EREG mRNAsi and mRNAsi in pan-cancer. The redder color indicates a stronger positive correlation, and the bluer color indicates a stronger negative correlation.

The Immune characteristics between high-risk and low-risk groups were demonstrated, including differences in Immune score, TIDE score and TMB (Figure 8A-C). Meanwhile, the correlation between CSC-related score and tumor stemness index (including mRNAsi, EREG-mRNAsi and mDNAsi) were presented in scatter plots (Figure 8D).

### Marker counts of mRNA, miRNA, protein, mutation, SCNV and drug sensitivity analysis between groups based on CSC-related score

**Figure 9:**
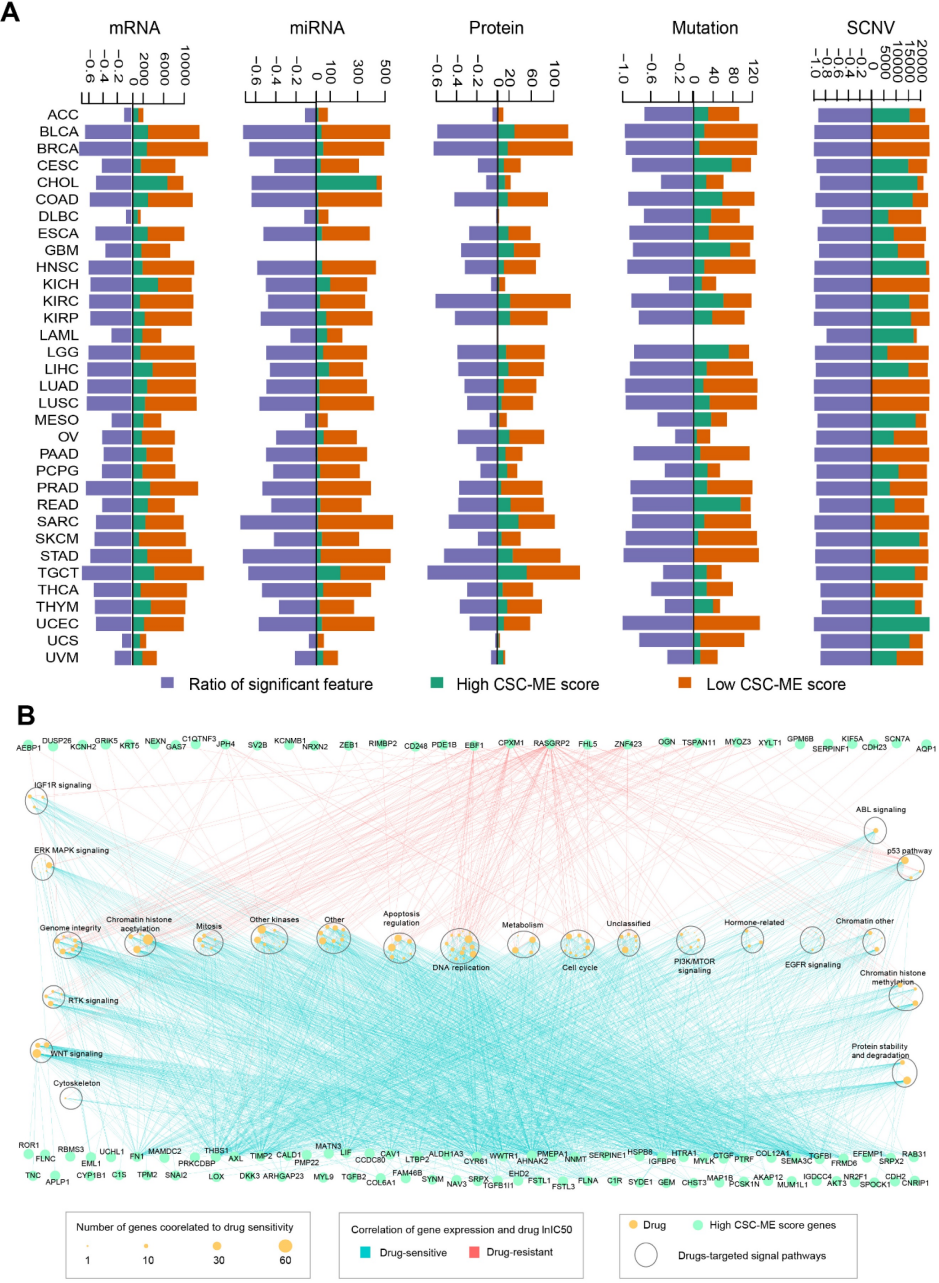
Marker counts of mRNA, miRNA, protein, mutation, SCNV and drug sensitivity analysis between groups based on CSC-related score. (A). Marker counts of mRNA, miRNA, protein, mutation and SCNV between groups based on CSC-related score. (B). mRNA markers, drug sensitivity and pathway enrichment analysis based on cell line data. The light blue line indicates a significant positive correlation between marker expression level and lnIC50 of the drug (R ≥ 0.3 and FDR ≤ 0.05), and the red line indicates a significant negative correlation between marker expression level and lnIC50 of the drug (R ≥ 0.3 and FDR ≤ 0.05). Green dots indicate marker genes with high CSC-related scores, yellow dots indicate drugs (size indicates the number of genes associated), and large oval boxes indicate pathways associated with drug targets.

We utilized a performance score algorithm in pan-cancer using logistic regression analysis, corrected for clinical factors (including confounding factors such as age), and screened genes for which confounding factors were balanced between the two groups (Methods and Materials). For mRNA, 19,793 marker genes were screened; For miRNA, 743 marker genes were screened. For protein level, 214 marker genes were screened; For the mutation level, 135 marker genes were screened; For SCNV, 1671 marker genes were screened. Then, we calculated and obtained differentially expressed marker genes at mRNA, miRNA, protein, mutation and SCNV levels according to the high-risk and low-risk groups (Methods and Materials) (Figure 9A). The Ratio of characteristic marker genes with significant differences between high and low groups was calculated.

For mRNA marker genes in the high CSC-related score group (genes present in at least 10 cancer types), we performed drug sensitivity analysis based on cell line drug response data. A total of 104 marker genes was associated with 141 significant drugs (|R| ≥ 0.3 and FDR ≤ 0.05, Figure 9B). In addition, we found that most of the mRNA marker genes showed a positive correlation with drug small molecules (R ≥ 0.3 and FDR ≤ 0.05), such as *FN1* and *FLNA* (Figure 9B). The corresponding signaling pathways of drug targets were explored, and a total of 23 were involved, such as DNA replication and WNT signaling (Figure 9B). In addition, we found that drugs related to chromatin histone acetylation pathway showed correlation with the most marker genes (Figure 9B).

## Discussion

CRC is one of the most prevalent malignant tumors worldwide, resulting in high morbidity and mortality^26^. Although CRC might be cured by radical surgery combined with chemo- and radiotherapy, drug resistance, recurrence and metastasis are still the main causes of CRC-associated mortality. Accumulating evidence showing CRC originates from cancer stem cells (CSCs)^27^,^28^. CSCs are capable of forming metastatic tumors owing to their proliferative capability^4^, and it is acknowledged that CSCs are the main reasons resulting in treatment resistance and disease recurrence in CRC^29^, which make them as promising therapeutic targets. In this study, we focused on the treatment planning and prognosis prediction value of CSC-related genes in pan-cancers especially CRC. We suggest that precise molecular subtyping of CSC-related genes would prospectively stratify CRC patients with different prognoses, TME infiltration patterns and therapeutic responses.

Among the 4 diverse molecular subtypes identified by consensus clustering based on the CSC-related genes, subtype 2 had significant better clinical outcome than subtype 3. To further elucidate the expression characteristics of the two subtypes, we performed differential expressed gene analysis, and a total of 1065 differential expressed genes were identified. The DEGs were intersected with CSC-related genes that connected with the lengh of survival by Univariate Cox regression analysis. Then LASSO regression analysis was performed, and a prognosis signature comprising 21 CSC-related genes in CRC was construted. The risk score of each patient was calculated and divided into high-risk and low-risk groups. The high-risk group had significant lower survival time than the low-risk group. With respect to immunotherapy, low-risk patients received better clinical benefits from ICIs when applying our signature to IMvigor210. Then we explored the application of the signature in pan-cancer. Patients with CNV exhibited significantly higher CSC-related scores compared to those without. Patients in high-risk group had better survival rate, immune score and TIDE score.

Previous research has partially elucidated the roles of CSC-related genes in cancer occurrence and development, as well as their potential as targets for cancer treatment. *GRB7*, growth factor receptor-bound protein 7, played an important role in MEKi resistance in CRC cells with KRAS mutations^30^. The overexpression of Protein Tyrosine Phosphatase Receptor Type N (*PTPRN*) promoted LUAD cell migration and the expression of EMT markers by influencing MEK/ERK and PI3K/AKT signaling^31^. Gastrin Releasing Peptide (*GRP*) is a kind of secretory protein and regulates numerous functions of gastrointestinal and central nervous system. *GRP* exerted mitogenic effect to accelerate proliferation of CRC and head and neck squamous cancer cells^32^. Fatty acid-binding protein 4 (*FABP4*), as a carrier protein for fatty acids, is widely expressed in adipocytes, macrophages, dendritic cells, and microvascular endothelial cells. It participates in lipid transport, metabolism, and intracellular signal transduction. *FABP4* may promote CRC progression related to epithelial-mesenchymal transition (EMT)^33^. Wnt signalling inhibitor *DKK1* Promotes tumor immune evasion and impedes Anti-PD-1 treatment^34^. The L1 cell adhesion molecule (*L1CAM*) promotes tumor growth and metastasis^35^. As a secretory protein, Inhibin βA (*INHBA*) is a member of the TGF-β superfamily. *INHBA* was aberrant overexpression in CRC tissues and closely related to the poor prognosis of CRC patients^36^. *TNS1* encodes cytoskeletal protein that maintains structural integrity and mediates signal transduction. Elevated *TNS1* expression in CRC cells had been revealed to increase cell proliferation and invasiveness^37^,^38^. *DPYSL4* is a member of the collapsin response mediator protein family, which is involved in cancer invasion and progression. *DPYSL4* plays a key role in the tumor-suppressor function of p53 by regulating oxidative phosphorylation and the cellular energy supply via its association with mitochondrial supercomplexes, possibly linking to the pathophysiology of both cancer and obesity^39^. *ISM1* promoted EMT and colon cancer cell migration and proliferation^40^. Different from the above, Fatty Acid Binding Protein 5 (*FABP5*) suppresses colorectal cancer progression^41^.

Taken together, through consensus clustering on CSC-related genes in CRC, 4 subtypes with diverse prognosis, immune infiltration levels and clinical characteristics were identified. By applying Univariate Cox regression analysis and LASSO analysis, a 21-gene CSC-related signature was constructed and validated in GEO cohorts of CRC patients. The model has prospective clinical implications for prognosis evaluation and and preferential use of ICIs in CRC. Furthermore, the expression levels of CSC-related genes in tumor cells are also related to prognosis, tumor mircroenvironment, treatment outcome, stemness score and the efficacy of different chemotherapy-related drugs in pan-cancer. These results thus provide a reference for future research on CSC-related genes as potential pan-cancer targets. Our study also has some limitations including lack of internal or external laboratorial validation of the newly developed prognostic model, as well as comparison with other existing prognostic markers/models, which is warranted in the future study.

## Supplementary Material

Supplementary figures.

## Abbreviations

CNV: copy number variation
COAD: colon adenocarcinoma
CR: complete remission
CRC: colorectal cancer
CSC: cancer stem cell
DEGs: differentially expressed genes
FDR: false discovery rate
GEO: gene expression omnibus
HR: hazard ratio
ICIs: immune-checkpoint inhibitors
LASSO: least absolute shrinkage and selection operator
OS: overall survival
PD: progressive disease
PFS: progression-free survival
PR: partial remission
ROC: receiver operating characteristic
SD: stable disease
TCGA: the cancer genome atlas
TIDE: tumor immune dysfunction and exclusion
TMB: tumor mutational burden
TME: tumor microenvironment
