# Supplementary material for: Construction of a Prognostic Model based on CSC-related Genes in Patients with Colorectal Cancer
Source: J Cancer. 2025 Apr 13;16(7):2375–87. doi: 10.7150/jca.108188 (PMC12036084; doi:10.7150/jca.108188)
Supplement: Supplementary file 1 — Supplementary figures. [file jcav16p2375s1.pdf]

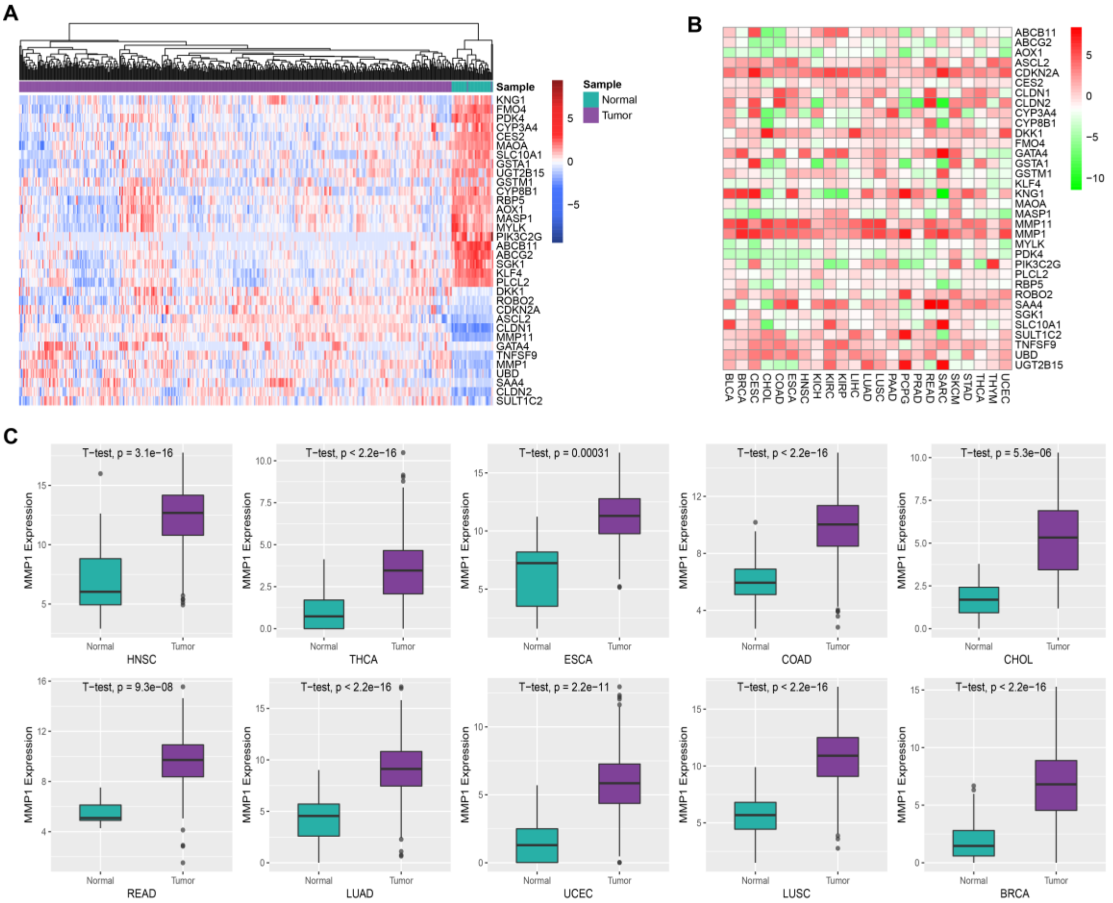

Figure S1 The differential expressed CSC-related genes in COAD and other cancers in TCGA. Thirty-four out of 206 selected CSC-related genes were differentially expressed in CRC tissues as compared with normal tissues (A), as well as in other cancers (B). (C) The expression of MMP1 in pan-cancer.

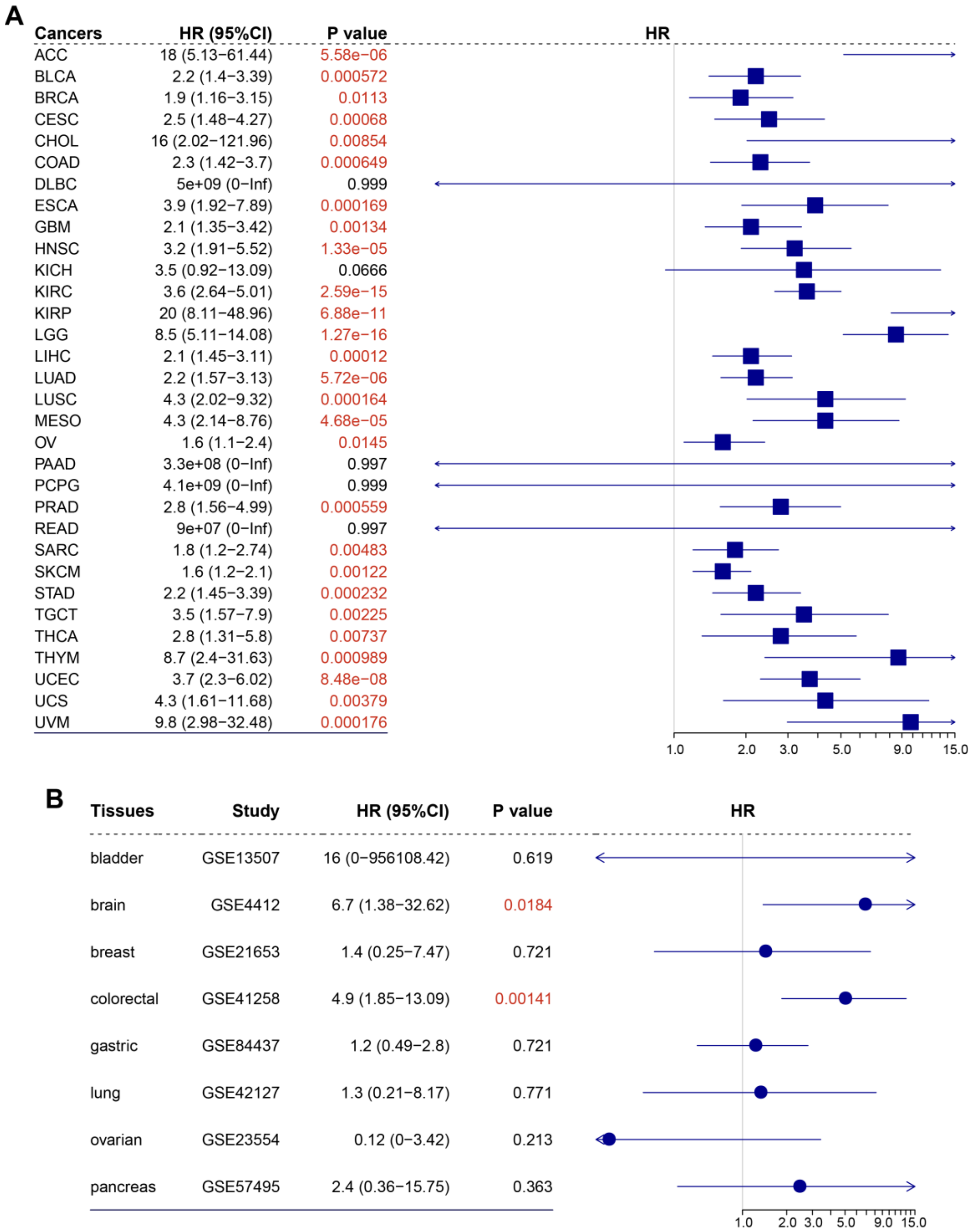

Figure S2 The differences in survival outcome were observed between subtypes classified based on the expression of DEGs in patients with diverse types of cancer in TCGA database (Figure S2A) and GEO datasets (Figure S2B)

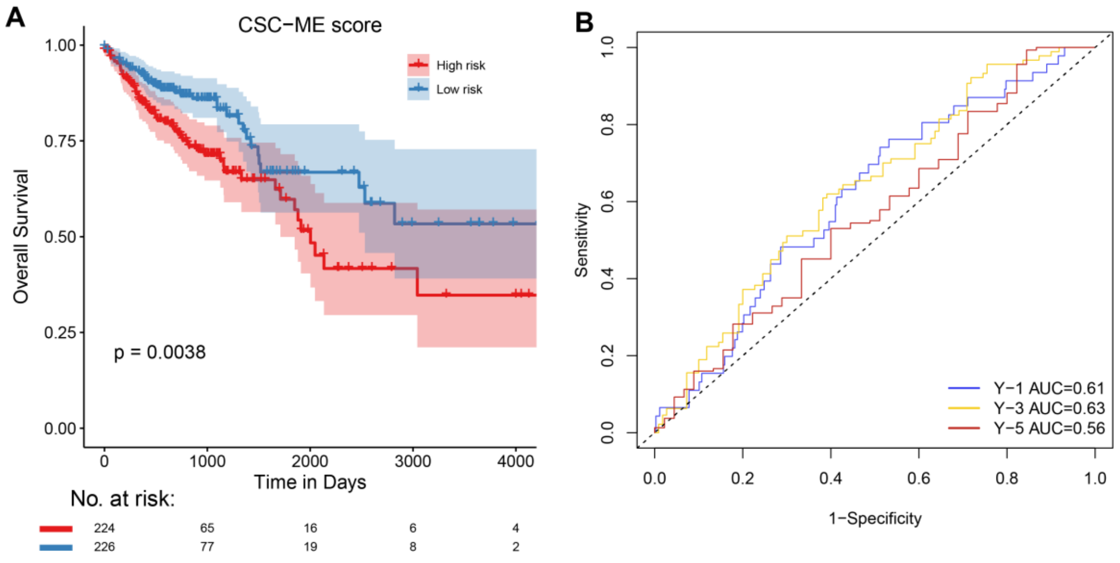

Figure S3 (A) Kaplan-Meier survival curve showing overall survival probability of high-risk or low-risk subgroups. (B) The 1-year, 3-year, and 5-year overall survival ROC curves are predicted by the signature

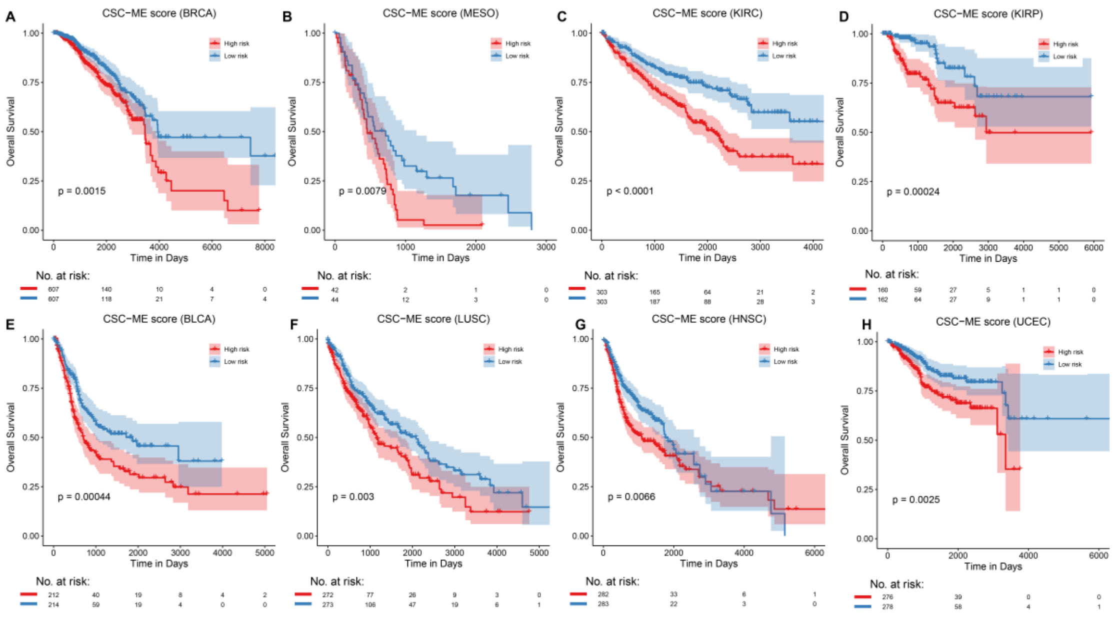

Figure S4 Survival curves (OS) of high-risk and low-risk groups in pan-cancer.
